# 

**Published:** 1867-10

**Authors:** 


### CONTENTS.

ORIGINAL COMMUNICATIONS.

Dental Nomenclature .......... 415
Management and best means of Preventing the Destruction of the Deciduous Teeth .......... 425
The Duty of the Hour .......... 430

PROCEEDINGS OF SOCIETIES.

Illinois State Dental Society .......... 435

EDITORIAL.

Prof. Peebles' Address .......... 448
Ohio College of Dental Surgery .......... 452
A Replanted Tooth .......... 453
Bibliographical .......... 454
Notice .......... 454
Tennessee Dental Association .......... 455
Personal—and Unselfish .......... 455
Obituary .......... 456

